# Radioprotective effect of epicatechin in cultured human fibroblasts and zebrafish

**DOI:** 10.1093/jrr/rrt085

**Published:** 2013-08-16

**Authors:** Hyang Ae Shin, Yoo Seob Shin, Sung Un Kang, Jang Hee Kim, Young-Taek Oh, Keun Hyung Park, Bum Hei Lee, Chul-Ho Kim

**Affiliations:** 1 Department of Otorhinolaryngology – Head and Neck Surgery, National Health Insurance Corporation Ilsan Hospital, 100 Ilsan Street, Ilsandong-Gu, Goyang, 410-719, Korea; 2 Department of Otolaryngology, School of Medicine, Ajou University, 164 Worldcup Street, Wonchon-Dong, Yeongtong-Gu, Suwon, 442–749, Korea; 3 Department of Pathology, School of Medicine, Ajou University, 164 Worldcup Street, Wonchon-Dong, Yeongtong-Gu, Suwon, 442–749, Korea; 4 Department of Radiation Oncology, School of Medicine, Ajou University, 164 Worldcup Street, Wonchon-Dong, Yeongtong-Gu, Suwon, 442–749, Korea

**Keywords:** radiation, epicatechin, radioprotection, fibroblast, zebrafish

## Abstract

Radiation-induced normal cell damage limits the delivery of high-dose radiation to targeted cancer. This study investigated the effect of epicatechin (EC), a minor component of green tea extracts, on radiation-induced cellular damage *in vitro* in primary cultured human fibroblasts and *in vivo* in a zebrafish model. Cell viability, proliferation and wound-healing efficacy, mitochondrial membrane potential, and reactive oxygen species (ROS) generation as well as changes in the signaling pathway related to apoptosis were investigated in fibroblasts. The therapeutic effects of EC were explored in a zebrafish model. EC increased clonogenic survival and restored the migration ability of the fibroblasts after irradiation. EC inhibited radiation-induced ROS generation, mitochondrial dysfunction and cell death. EC significantly reduced the expression of p-JNK, p-38, and cleaved caspase-3 compared with their significant increase after radiation treatment. EC attenuated the radiation-induced embryotoxicity in a zebrafish model. These results suggest that EC represents an effective means of reducing cellular damage and facilitating wound healing after radiation exposure.

## INTRODUCTION

Radiotherapy has become increasingly important for treatment of various types of cancer [1, 2]. However, radiation therapy for any cancer treatment faces a crucial dilemma: delivering a sufficient radiation dose for tumor control while limiting normal tissue damage as much as possible. Exposure of normal tissue to radiation can cause both acute and chronic toxicities including dermatitis, xerostomia or mucositis, and this can result in not only a cessation of the intended therapy, but also a decrease in quality of life for the patient. There has been a lot of effort to reduce the radiation toxicities, mainly by focusing on technological improvements in radiation delivery such as conformal radiotherapy, intensity-modulated radiotherapy, image-guided radiotherapy, and proton radiotherapy [3–6]. Such technological advances enable physicians to deliver sufficient radiation to the target lesion with less exposure to normal tissue. An alternative method for reducing normal tissue damage is the use of radiation protective agents prior to or shortly after radiation exposure [7, 8]. Although a large number of compounds have shown promise as radioprotectors in laboratory studies, most of them have failed to reach the preclinical stage because of toxicity and side-effects [8–10]. Therefore, many researchers have focused on herbal or medicinal ingredients as radioprotective agents because of the advantage of their having reduced side-effects and easier accessibility.

Green tea consumed in a balanced and controlled diet improves anti-oxidative status and can protect against oxidative damage [11]. Beneficial activities attributed to green tea extracts and/or constituents include antibacterial [12], antiviral [13], antioxidative [14], antitumor [15], and antimutagenic [16] activities. Furthermore, green tea extract can scavenge nitric oxide (NO) and superoxide anions (O_2_^−^) very effectively [17]. In a previous study, we documented that epicatechin (EC), a component of green tea, prevents cisplatin- and radiation-induced, ROS-mediated ototoxicity and prevents changes in mitochondrial membrane potential [18]. EC is among the important constituents responsible for the protective and antioxidant effects exhibited by green tea and is also active in lessening ionizing radiation-induced damage to DNA [19, 20]. However, the effect of EC as a radioprotective agent has not been investigated. The goal of the present study is to investigate the *in vitro* effects of EC on radiation-induced inhibition of cell proliferation, migration and viability in primary human fibroblasts, and in addition, the *in vivo* effect in zebrafish embryos. Moreover, the associated signaling mechanisms, specifically those involving mitogen-activated protein kinase (MAPK) and caspase-3, were also studied.

## MATERIALS AND METHODS

### Cell line and irradiation conditions

The fibroblast cell line that we used in this study was established by primary culture from human oral mucosa. Cell lines were cultured in RPMI1640 (Gibco, Grand Island, NY, USA) supplemented with 10% (v/v) fetal bovine serum at 37°C in a humidified atmosphere under 95% air, 5% CO_2_. The culture medium was changed twice per week. Cell morphology was observed using light microscopy, and all cells were used after less than five passages. Cells were prepared in 24-well plates and irradiated with single doses of 20 Gy. A single 20 Gy dose was delivered by opposed photon beams at a rate of 2 Gy/min using the LINAC, 6MV (21EX; Varian, Palo Alto, CA, USA)

### Cell viability assay

Fibroblasts were pretreated with EC for 1 h, and then washed twice with cold PBS and prepared in 24-well plates. A single dose of 20 Gy was applied. To determine cell viability, the primary cultured fibroblasts were seeded on 96-well plates at densities of 5 × 10^3^ cells/well in 1 ml complete medium after being exposed to radiation (20 Gy), various concentrations of EC (0–200 µM), or radiation plus EC*.* EC was purchased from Sigma-Aldrich (St Louis, MO, USA). This product is soluble in water (5 mg/ml) and intraperitoneal (mouse) LD50 is 1000 mg/kg. At 5 days after irradiation, 3-(4,5-dimethyl-thiazol-2-yl)-2. 5-diphenyl-2H-tetrazolium bromide (MTT; Sigma-Aldrich) was added to 40 µl of the cell suspension for 4 h. After three washes with phosphate buffered saline (PBS, pH 7.4), the insoluble formazan product was dissolved in dimethyl sulfoxide (DMSO). The optical density (OD) of each culture well was measured using a microplate reader (Bio-Tek, Winooski, VT, USA) at 540nm.

### Colony-forming assay

To determine long-term effects, cells were untreated or treated with 50 µM EC for 3 h after being exposed to a single dose of radiation (20 Gy) in 24-well plates. After being rinsed with fresh medium, cells were allowed to grow for 3 days to form colonies, which were stained with 4% crystal violet (Sigma-Aldrich). More than 2 mm of the cells were enumerated.

### Wound-healing assay

Investigation of the cell migration capability of the primary cultured fibroblasts was performed using a wound-healing assay, as previously described [21]. Briefly, fibroblasts were grown to confluent monolayers. The monolayers were wounded by scratching the surface as uniformly as possible with a 1-ml pipette tip. The cells were pretreated with various concentrations of EC (0, 50 or 100 µM) for 1 h. Then the cells were treated with radiation (20 Gy), and after 1 h the culture media containing EC was replaced with pure RPMI1640. The images of the wound area were captured on Day 0 (day of scratching) and Day 2 using an Olympus SC 35 camera (Tokyo, Japan) connected to an inverted microscope. The migration rate of the fibroblasts was determined by measuring the percent closure area. The following formulae were used: percent closure (%) = (migrated cell surface area ÷ total surface area) x 100; migrated cell surface area = length of cell migration (mm) x 2 x length; total surface area = 2.4 mm x length. For each concentration of EC and each time-frame, the experiments were repeated three times.

### Mitochondrial membrane potential assay

The mitochondrial membrane potential (MMP) of intact cells was measured by flow cytometry with the lipophilic cationic probe 5,5 V,6,6 V-tetrachloro-1,1 V 3,3 V-tetra ethylbenzimidazolcarbocyanine iodide (JC-1; Molecular Probes, Eugene, OR, USA). The culture medium was briefly removed from the adherent fibroblasts, and the cells were rinsed with PBS. Cell monolayers were incubated with RPMI1640 (Gibco) and 5 mg/ml JC-1 at 33°C for 20 min. The cells were subsequently washed twice with cold PBS and trypsinized, and then treated with EC (50 µM) only, radiation only (20 Gy), or radiation plus EC. Cell pellets were then resuspended in 500 µl of PBS. The change in MMP was measured by flow cytometry (FACScan, BD Biosciences, San Jose, CA, USA).

### Measurement of intracellular ROS production

Intracellular generation of ROS was quantified using 5-(and 6)-carboxyl-2',7'-dichlorodihydro fluorescein diacetate (DCFDA; Molecular Probes). For the assay, fibroblasts were cultured overnight in 6-well plates. Before irradiation, cells were pretreated with EC (50 µM) for 1 h and washed twice with cold PBS, then treated with 20 Gy of radiation. The cells were incubated in the dark with 10 µM DCFDA in serum-free medium for 10 min at 33°C for 24 h. An oxidative burst (hydrogen peroxide, H_2_O_2_) was detected using a FACScan flow cytometer (BD Biosciences) with excitation and emission settings at 488 and 530 nm, respectively.

### Western blot assay

Fibroblasts were pretreated with EC (50 µM) for 1 h, and then the cells were washed twice with cold PBS. Subsequently, some cells were irradiated with 20 Gy or and some cells were not. Total proteins were extracted using the ProteoExtract^®^ Subcellular Proteome Extraction Kit (Calbiochem, La Jolla, CA, USA) following the manufacturer's instructions. Protein concentrations were measured using the BCA assay (Pierce, Rockford, IL, USA). The proteins were separated by electrophoresis on 12% and 10% sodium dodecyl sulfate polyacrylamide (SDS) gels. An equal amount of protein (10 µg) was loaded in each lane. After electrophoresis, the proteins were transferred onto polyvinylidene difluoride (PVDF) membranes. The membrane was blocked in Tris-Buffered Saline Tween-20 (TBST) containing 5% non-fat milk for 1 h, followed by overnight incubation at 4°C with primary antibodies. All primary antibodies were purchased from Cell Signaling Technology (Danvers, MA, USA). After washing the membrane extensively, incubation with horseradish peroxidase-conjugated secondary antibody (1:1000, Cell Signaling Technology) was performed for 1 h at room temperature. Protein bands on the blots were visualized by ECL Plus Western Blot detection reagents (Amersham Pharmacia Biotech, Piscataway, NJ, USA).

### Radioprotective effects on zebrafish

Freshly fertilized embryos (6 h postfertilization) were exposed to radiation (20 Gy) and to 200 µM of EC. The embryos were pretreated with EC for 1 h, after which the eggs were washed twice with sea salt 1X. Subsequently, the embryos were seeded in 96-well plates at densities of 10 embryos/well in 1 ml sea salt 1X and treated with radiation. Four days after exposure, survival of embryos was assessed visually at 24-h intervals up to 7 dpf (days postfertilization) by light microscopy. Mortality was identified by missing heartbeat, coagulation of the embryos, a non-detached tail and failure to develop somites. The morphology was assessed visually using an AXIO vert200 light transmission microscope (Carl Zeiss, Göttingen, Germany) at a magnification of ×60–100. Representative images were recorded using Axiovision software (Carl Zeiss).

### Statistical analyses

All values were expressed as mean ± standard deviation, and statistical analysis was performed by the Kruskall–Wallis Test and the Mann–Whitney U Test. The survival rates were evaluated using the Kaplan Meier method (SPSS, version 17, Chicago, IL, USA). A *P*-value < 0.05 was regarded as statistically significant.

## RESULTS

### Epicatechin increased the viability and migration capability of irradiated primary cultured fibroblasts

**Fig. 1.**
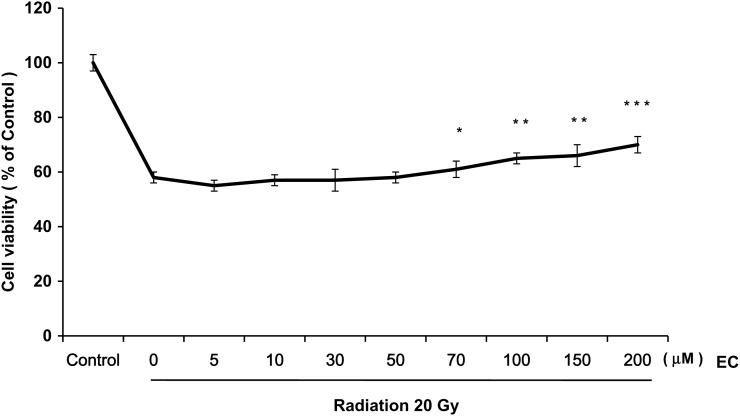
Effect of epicatechin (EC) on viability of the primary cultured fibroblasts after irradiation. The primary cultured fibroblasts were exposed to a single dose of radiation (20 Gy) and various concentrations of EC (0–200 µM). At 5 days after irradiation, cell viability was measured by MTT assay. The data represent the mean ± SD of three independent experiments. *P < 0.05, **P < 0.01, ***P < 0.001, compared with radiation alone.

**Fig. 2.**
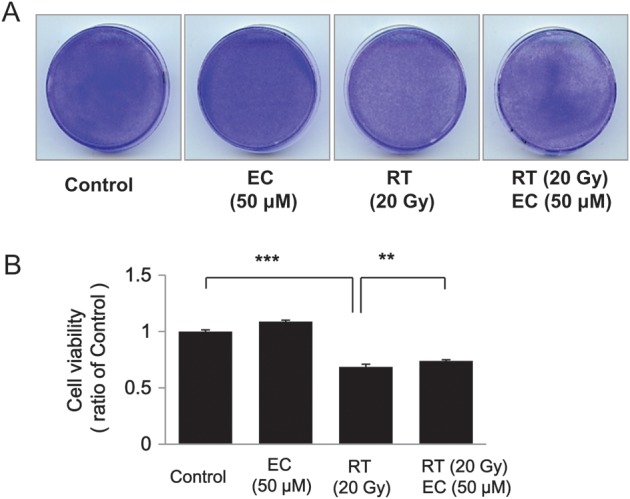
Radioprotective effect of epicatechin (EC) on the ability of the primary cultured fibroblasts to form colonies on soft agar. The primary cultured fibroblasts were treated with 50 µM EC for 3 h and then incubated for 3 days to form colonies. After 4% crystal violet staining, more than 2 mm of the colonies were counted. (**A**) Representative dishes by colony-forming assay. (**B**) Irradiation significantly decreased colonogenic survival in the fibroblasts. EC increased colonogenic survival after radiation treatment. The data represent the mean ± SD of three independent experiments. **P < 0.01, ***P < 0.001 compared with radiation alone.

**Fig. 3.**
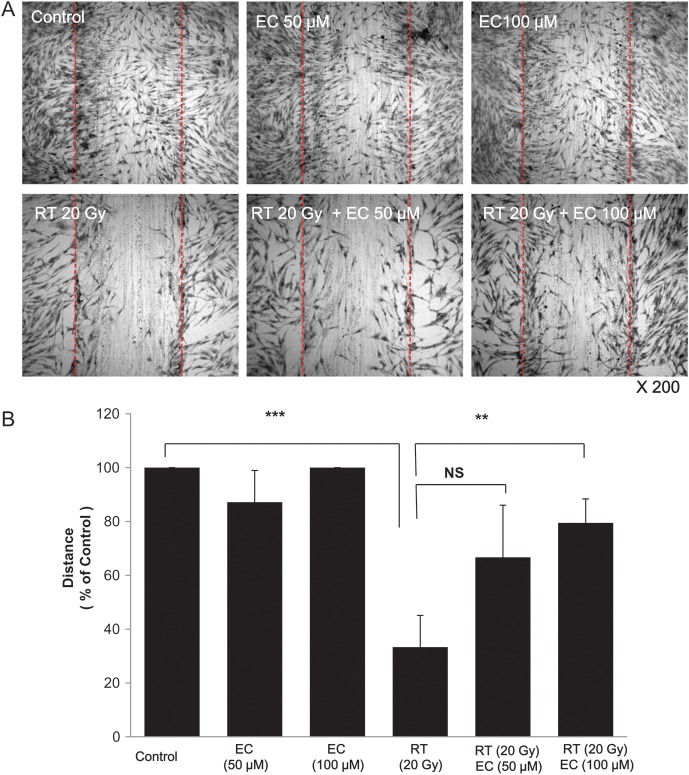
Epicatechin (EC) restores cell proliferation and migration, which were decreased after irradiation. Investigation of cell migration capability after irradiation treatment was performed with the wound-healing assay. (**A**) Confluent monolayers of the primary cultured fibroblasts were wounded by scratching the surface as uniformly as possible with a 1-ml pipette tip. After treatment/no treatment with a single 20-Gy dose of irradiation, the cells were cultivated for another 24 h. (**B**) Percent closure of wound areas was measured. EC treatment significantly restored cell proliferation and migration on 100 µM of EC. The data represent the mean ± SD of three independent experiments. **P < 0.01, ***P < 0.001.

As shown in Figs 1 and 2, radiation decreased the viability of the primary cultured fibroblasts. We examined the effect of various concentrations of EC on the radiation-treated cells and discovered that EC significantly protected the primary cultured fibroblasts from radiation-induced cytotoxicity in a dose-dependent manner (Fig. 1). To analyze this suppressive effect of EC on the survival of the primary cultured fibroblasts, we performed a colony-forming assay. As shown in Fig. 2, the pretreatment with 50 µM EC before irradiation increased the survival rates of irradiated cells. Statistical significance (*P* < 0.05) was seen on the survival rates of the EC-treated cells compared with those of the untreated cells after irradiation at 20 Gy (Fig. 2). The wound-healing assay was done to determine the migration capability of the fibroblasts. EC restored the proliferation and migration ability (Fig. 3). Proliferation and migration were restored up to approximately 80% at a 100-μM concentration of the EC.

### Epicatechin inhibited radiation-induced decrease of mitochondrial membrane potential (MMP)

**Fig. 4.**
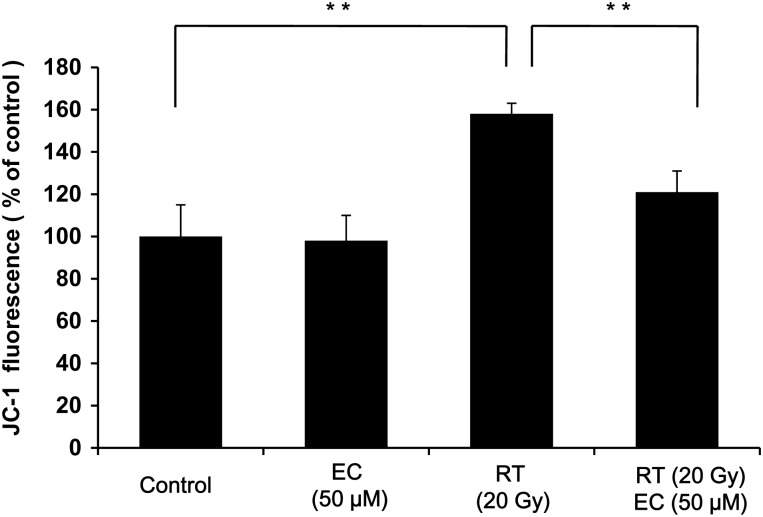
Epicatechin (EC) inhibited the radiation-induced decrease of MMP in the primary cultured fibroblast. The cells were treated with radiation (20 Gy) and EC (50 µM) and stained with JC-1. The MMP change was objectively measured using FACScan flow cytometry. The data represent the mean ± SD of three independent experiments. **P < 0.01.

MMP can be used as an indicator of mitochondrial damage. JC-1 is a lipophilic, cationic dye that can selectively enter into mitochondria and reversibly change color from green to red as the membrane potential increases. Control and EC-only treated cells maintained a high MMP indicated by the red fluorescence of the JC-1 dye. However, irradiation clearly increased the green color, indicating a loss of MMP (*P* < 0.01), and EC restored red fluorescence (Fig. 4). Therefore, EC inhibited radiation-induced changes in the MMP.

### Epicatechin inhibited intracellular ROS generated by radiation

**Fig. 5.**
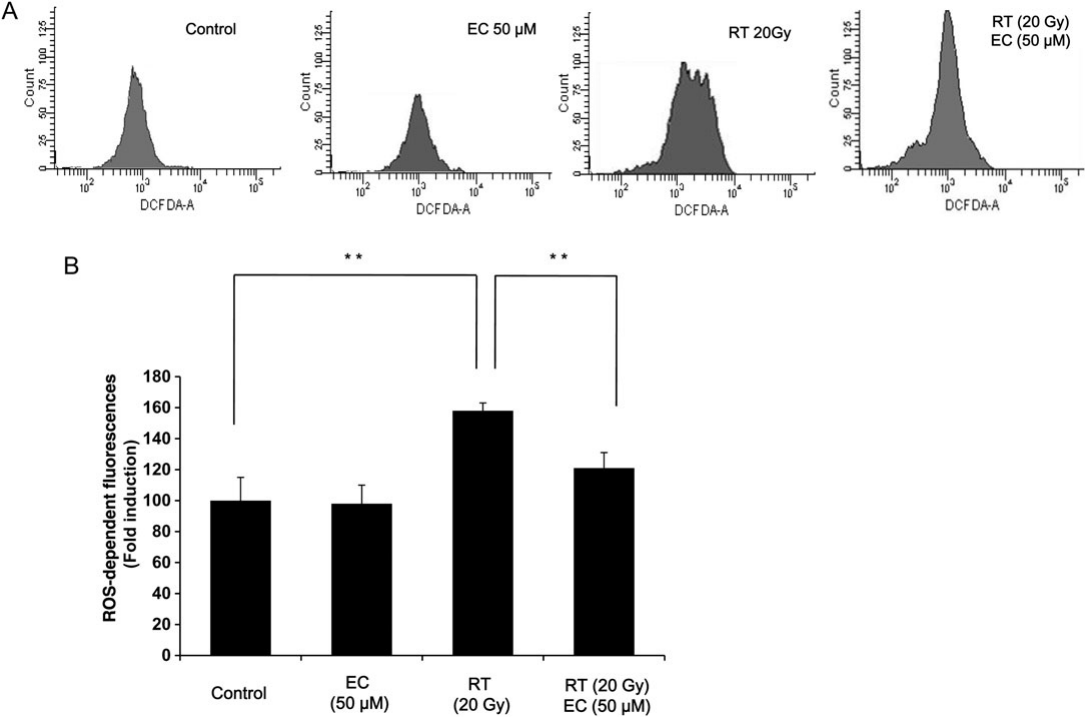
Effect of EC on intracellular ROS generated by radiation in primary cultured fibroblasts. (**A**) For measurement of ROS generation, the primary cultured fibroblasts were cultured overnight in 6-well plates and then treated with 20 Gy of radiation in the presence or absence of EC (50 µM). The level of intracellular ROS was measured by FACScan flow cytometry using the peroxide sensitive fluorescent probe, DCFDA. (**B**) The results (mean ± SD) were calculated as a percent of the control group (not exposed to radiation). The data represent the mean ± SD of five independent experiments. **P < 0.01.

We next investigated the effect of EC on radiation-induced ROS generation. Cells were treated with 20 Gy of radiation, and the level of intracellular ROS was monitored by FACScan flow cytometry with the peroxide sensitive fluorescent probe, DCFDA. Treatment with radiation significantly increased the generation of intracellular ROS, and this was significantly inhibited by EC (Fig. 5A, B).

### Inhibition of MAPK activity by epicatechin rescued the primary cultured fibroblasts from radiation-induced cytotoxicity

**Fig. 6.**
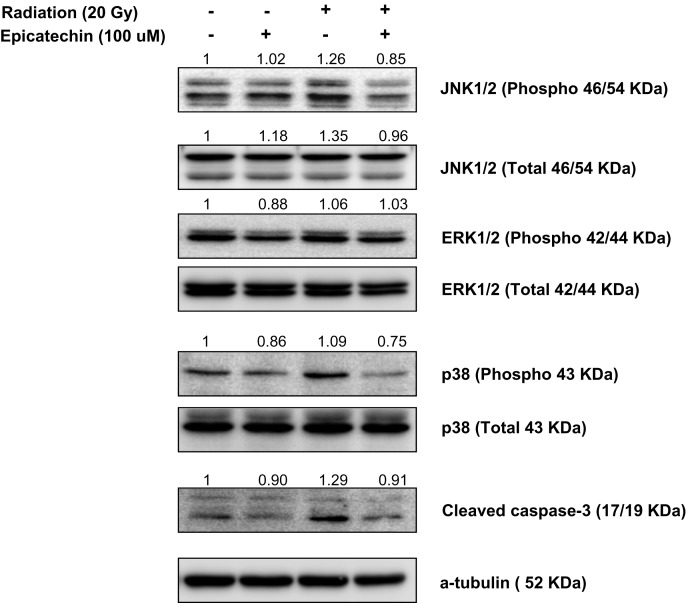
Epicatechin (EC) inhibited radiation-induced apoptosis of the primary cultured fibroblasts via inhibition of MAPKs activation and caspase-3-dependent pathway. The cells were pretreated with 50 µM of EC followed by the addition of 20 Gy radiation. Cell lysates were collected, electrophoresed through an SDS–polyacrylamide gel, and subjected to immunoblot analysis with antibodies against p38, JNK, ERK, and cleaved caspase-3.

To elucidate the mechanism underlying the activity of radiation and EC, we evaluated the radiation and EC-induced changes on gene expression. We evaluated gene-related apoptosis of p38, JNK, ERK, and cleaved caspase-3. A representative western blot is shown in Fig. 6; the results obtained confirmed increased expression of p-JNK, p38, and cleaved caspase-3 after radiation treatment. EC reduced expression of p-JNK, p38, and cleaved caspase-3 that had been increased after radiation treatment (Fig. 6). These results suggest that EC blocked radiation-induced apoptosis via downregulation of JNK, p-38 and cleaved caspase 3 in primary cultured fibroblasts.

### Epicatechin protects zebrafish embryos from radiation cytotoxicity

**Fig. 7.**
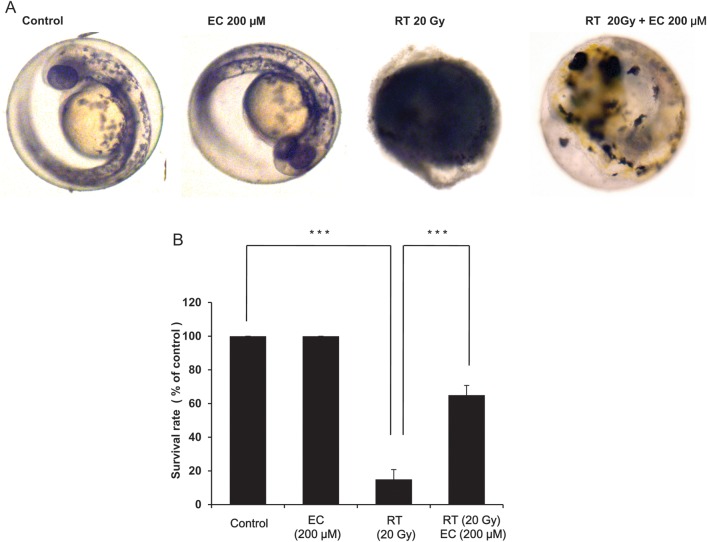
Protective effect of epicatechin (EC) against radiation-induced toxicity in zebrafish embryos. Embryos were exposed to radiation (20 Gy) at 6 h postfertilization in the absence and presence of EC (200 µM). (**A**) Irradiation-induced malformation. (**B**) Survival rate of zebrafish embryos. The data represent the mean ± SD of three independent experiments. ***P < 0.001.

To determine the *in vivo* radioprotective effect of EC, we exposed zebrafish embryos at 6 h postfertilization to 20 Gy of radiation plus EC (200 µM) and investigated the effects of this treatment on their morphologic appearance and survival up to 7 dpf of development. Radiation decreased survival rates of the zebrafish embryos. Fewer morphologic changes were induced in the EC-treated zebrafish embryos (Fig. 7A). Furthermore, survival rate was significantly improved when the zebrafish embryos were treated with EC (Fig. 7B, *P* < 0.001).

## DISCUSSION

Conventional radiation therapy for various cancers consists of high doses of radiation that inevitably lead to the development of severe acute side-effects. New radiotherapy regimens such as accelerated radiation therapy [22] and the combination of radiotherapy with new molecular targeted drugs [23] have improved the therapeutic index but have also increased acute toxicity, which may lead to tissue necrosis. Therefore, understanding and conceiving a way to ameliorate the normal tissue damage is a very important issue in radiation oncology. Because the exposure of cells to irradiation causes free radical formation, which can break both DNA and the cytoplasmic organelles, such as mitochondria or the endoplasmic reticulum, a reduction of oxygen free radicals has been proposed as a major means for normal tissue protection [10]. Meanwhile, many researchers have focused on commonly used medicinal plants and have shown their antioxidant properties and potentials in preventing radiation damage [24–28]. Of the several plausible materials, we previously showed the otoprotective effect of the green tea extract epicatechin, which was revealed to prevent ototoxicity induced by radiation and cisplatin via the reduction of ROS generation [18, 20, 29]. Based on this finding, we conceived the idea that EC could serve as a radioprotectant in other types of cells as well. The aim of the present study was to determine the effectiveness of EC on radiation-induced cellular damage *in vitro* in cultured human fibroblasts and *in vivo* in a zebrafish model.

In this study, radiation-induced cell death was demonstrated by looking at cell viability and colony-forming capability in primary cultured fibroblasts. EC successfully reversed these radiation-induced phenomena. EC restored the migration ability of the fibroblast, which was decreased after irradiation. Moreover, EC decreased ROS that were generated in response to radiation. These findings suggest that radiation-induced cytotoxicity is mediated by ROS and that EC may prevent toxicity by decreasing oxidant-mediated cell damage in fibroblasts. JNK, p38 in MAPKs, and cleaved caspase were activated by irradiation and EC treatment reversed these. EC exerts a protective effect on radiation-induced cytotoxicity and these effects were confirmed using an *in vivo* zebrafish model.

We used primary cultured fibroblasts in this investigation, because fibroblasts are abundant in the gastrointestinal tract mucosa and dermis which are well known to be closely connected with wound healing or consequent fibrosis [30, 31]. Therefore, we speculated that an *in vitro* fibroblast–radiation injury system could well reflect conditions in general radiation toxicity such as radiation dermatitis or mucositis. In addition, we chose the concentration of 50 µM EC in most of our *in vitro* experiments because of our previous experience. We previously reported a protective effect of EC in radiation-induced toxicity in HEI-OC1 cells [20, 29]. In these reports, 50 µM EC showed a successful protective effect against irradiation. Furthermore, in preliminary experiments, 50 µM EC was also revealed to have a radioprotective effect in human fibroblasts.

Although the exact relationship between ROS and other cell death events is still unclear [32], ROS is believed to play a key role in the promotion of apoptosis by affecting mitochondrial permeability, release of cytochrome c, activation of p53, and the action of caspases [33]. Based on a ROS-dependent mechanism of radiation-induced cytotoxicity, various antioxidant strategies can be considered. Previously, we demonstrated that radiation-induced apoptosis may be mediated by ROS production and successfully inhibited by EC through blocking ROS generation, inhibiting changes in the mitochondrial membrane potential, and inhibition of MAPK in auditory hair cell lines [20]. EC also attenuated the radiation-induced embryo-toxicity and protected against radiation-induced loss and changes of auditory neuromasts in zebrafish. In this investigation, radiation caused an increase of intracellular ROS, disruption of mitochondrial permeability, and consequent cell death in primary cultured fibroblasts, and these events were successfully reversed by EC pretreatment.

Irradiation results in the activation of multiple MAPK pathways simultaneously in a cell-type dependent manner [34]. In this investigation, EC significantly reduced expression of p-JNK, p38, and cleaved caspase-3 that were increased after radiation treatment. In addition, pharmacological inhibition of p38 and JNK also effectively prevented the cell death in radiation-treated fibroblasts (data not shown). These results suggest that EC blocked radiation-induced apoptosis via downregulation of JNK and p38 in primary cultured fibroblasts.

Tea, which is one of the most popular beverages globally, has an antioxidant activity [35]. Tea preparations have been shown to trap ROS such as O_2_, single oxygen, hydroxyl radical, peroxyl radical, NO, nitrogen dioxide and peroxynitrite, reducing their damage to the lipid membrane, proteins, and nucleic acids in cell-free systems [11]. EC is a minor constituent (4–6%) of tea polyphenols and is relatively inactive. Thus, few studies have probed the antioxidant effects of EC. However, EC has the capacity to quench OH^−^ radicals almost 100 times more effectively than mannitol, a typical OH^−^ radical scavenger [36]. Moreover, EC possesses free radical scavenging (IC_50_ = 1.5 µg/ml), potent hydrogen peroxide (IC_50_–11.18 µg/ml) and superoxide anion scavenging (IC_50_–1.64 µg/ml) activities [37]. EC inhibits spontaneous and photo-enhanced lipid peroxidation and protects phospholipid bilayers against lipid peroxidation induced by 2,2'-azobis [2-amidinopropane], a water-soluble radical initiator [38]. Finally, EC reduces cell membrane damage by lowering lipid peroxidation and restoring the activity of antioxidant enzymes such as superoxide dismutase and catalase [38]. For clinical application of any compound as a radioprotective agent, absolute certainty about the protection factors for tumor and normal tissue is mandatory to avoid unacceptable clinical risk. As EC is a dietary ingredient and has been used by humans for millenia, this compound should be non-toxic and safe. Although a large number of compounds have shown promise as radioprotectors in laboratory studies, most of them failed to reach the preclinical stage because of toxicity and side effects. In this study, EC administration was not toxic to zebrafish embryos, as neither viability nor gross morphology was adversely affected. In addition, EC given before radiation conferred a significant survival advantage to zebrafish embryos exposed to 20 Gy. Therefore, EC could be a plausible radioprotector or a radiation mitigator agent, which may help to reduce radiation damage in individuals unintentionally exposed through accidents who can only be treated after the exposure has occurred. Further studies with tumor-bearing animals are needed to examine whether EC has a preferential radioprotective action for normal tissues over tumor tissues. However, it has been demonstrated that administration of vitamin E and epigallocatechin, a compound related to EC and occurring in green tea extract, significantly inhibits tumor growth rate [39]. The same study also reported that administration of these antioxidants reduced deleterious effects, due to radiation exposure, in normal tissues of tumor-bearing mice [39].

## CONCLUSION

In conclusion, we demonstrated that EC, one of the tea catechins, protected the primary cultured fibroblasts *in vitro* from radiation by blocking ROS generation and inhibition of MAPKs. Moreover, EC is an effective means of rescuing the zebrafish embryo from irradiation. Although larger numbers of animals and clinically relevant fractionation schemes are necessary to confirm the effects of EC, these results suggest the potential of EC to inhibit radiation-induced cytotoxicity.

## FUNDING

This work was supported by a National Research Foundation of Korea (NRF) grant funded by the Korean Government (MEST) (2010-0012821).
